# Auricular Gouty Tophi: A Rare Presentation in an Uncommon Site

**DOI:** 10.7759/cureus.81035

**Published:** 2025-03-23

**Authors:** Lee Kuang Joo, Nor Shahida Abd Mutalib, Farveen Marican Abu Backer, Nurul Syeha Abdull Rasid, Hawairy Samsuri, Noor Dina Hashim

**Affiliations:** 1 Otorhinolaryngology - Head and Neck Surgery, Universiti Kebangsaan Malaysia Medical Centre, Kuala Lumpur, MYS; 2 Otorhinolaryngology - Head and Neck Surgery, Hospital Sultan Abdul Halim, Sungai Petani, MYS; 3 Pathology, Hospital Sultan Abdul Halim, Sungai Petani, MYS

**Keywords:** auricle, gout, gouty tophi, hyperuricemia, monosodium urate (msu) crystal

## Abstract

Unlike the intense redness and painful swollen joint seen in acute gouty arthritis, classically affecting the first metatarsophalangeal joint called podagra, painless gouty tophi nodules can be found in any soft tissue throughout the body. While they frequently affect areas like the toes, fingers, and olecranon bursae, it is rare to see monosodium urate crystal deposits in the facial region, particularly the ear. This case highlights a patient with uncontrolled hyperuricemia, presenting with an uncommon manifestation of gouty tophi on the auricle. The diagnosis of auricular gouty tophi should be the primary consideration in patients with a nodular auricular mass, whitish material, uncontrolled hyperuricemia, and multiple gouty tophi. Although clinical history, examination findings, and biochemical investigations provide important diagnostic clues, definitive confirmation necessitates surgical excision and histopathological analysis.

## Introduction

Gout is a metabolic disease characterized by a high blood uric acid level, resulting in the formation of monosodium urate crystals deposited in the body [1]. Over time, it progresses through four distinct sequences: asymptomatic hyperuricemia, acute gout, intercritical gout, and advanced or chronic tophaceous gout [2].

The development of gouty tophi is influenced by the level of uric acid in the blood and the duration of chronic gout. Uric acid crystal forms when the blood uric acid concentration exceeds 7 mg/dL (~416 umol/L) [3,4]. In the case of chronic, undertreated gout, gouty tophi generally develops after 5-10 years [5]. Gouty tophi can form in any joint or soft tissue in the body. It appears clinically as a palpable nodule containing uric acid crystals and chronic granulomatous tissue within subcutaneous tissues, joints, or tendons [6].

Gout typically begins with the onset of single-joint arthritis. Over time, however, gouty tophi can develop in multiple parts of the body, with 25% of patients forming these deposits after 20 years [7]. Common sites of tophi formation are in the areas of mechanical stress, such as adjacent to the first metatarsophalangeal (MTP) joint, the Achilles and patellar tendons, and the olecranon and prepatellar bursae [8,9]. In the head and neck region, tophi can also appear in more cosmetically concerning locations, such as the auricular appendages and the tip of the nose [10]. However, reports of gouty tophi forming on the auricle are rare [11]. Here, we are reporting a case of a 66-year-old male with uncontrolled hyperuricemia presenting with a gouty tophi mass on his right auricle.

## Case presentation

A 66-year-old man presented to the otorhinolaryngology clinic with a painless mass over the lateral surface of his right auricle, seeking to have it removed. The mass had gradually increased in size over 10 years. He denied any associated ear pain, discharge, hearing loss, tinnitus, or vertigo. Additionally, he had no history of trauma or insect bites prior to the onset of the mass. His medical history was notable for diabetes mellitus, hypertension, dyslipidemia, and a 20-year history of gout. Unfortunately, he had been non-compliant with his prescribed urate-lowering medication, allopurinol.

**Figure 1:**
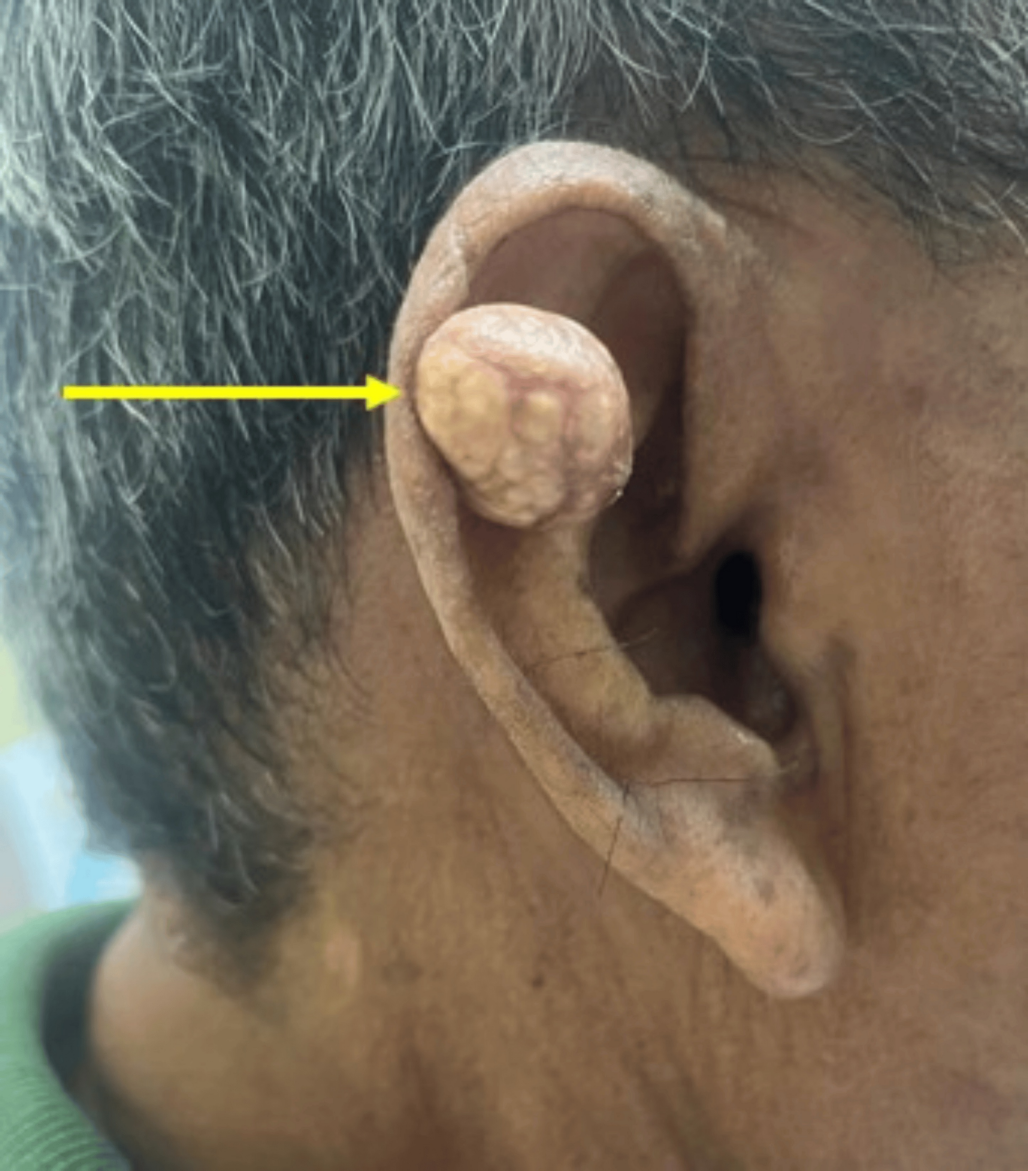
Right auricular nodular mass with whitish material within

**Figure 2:**
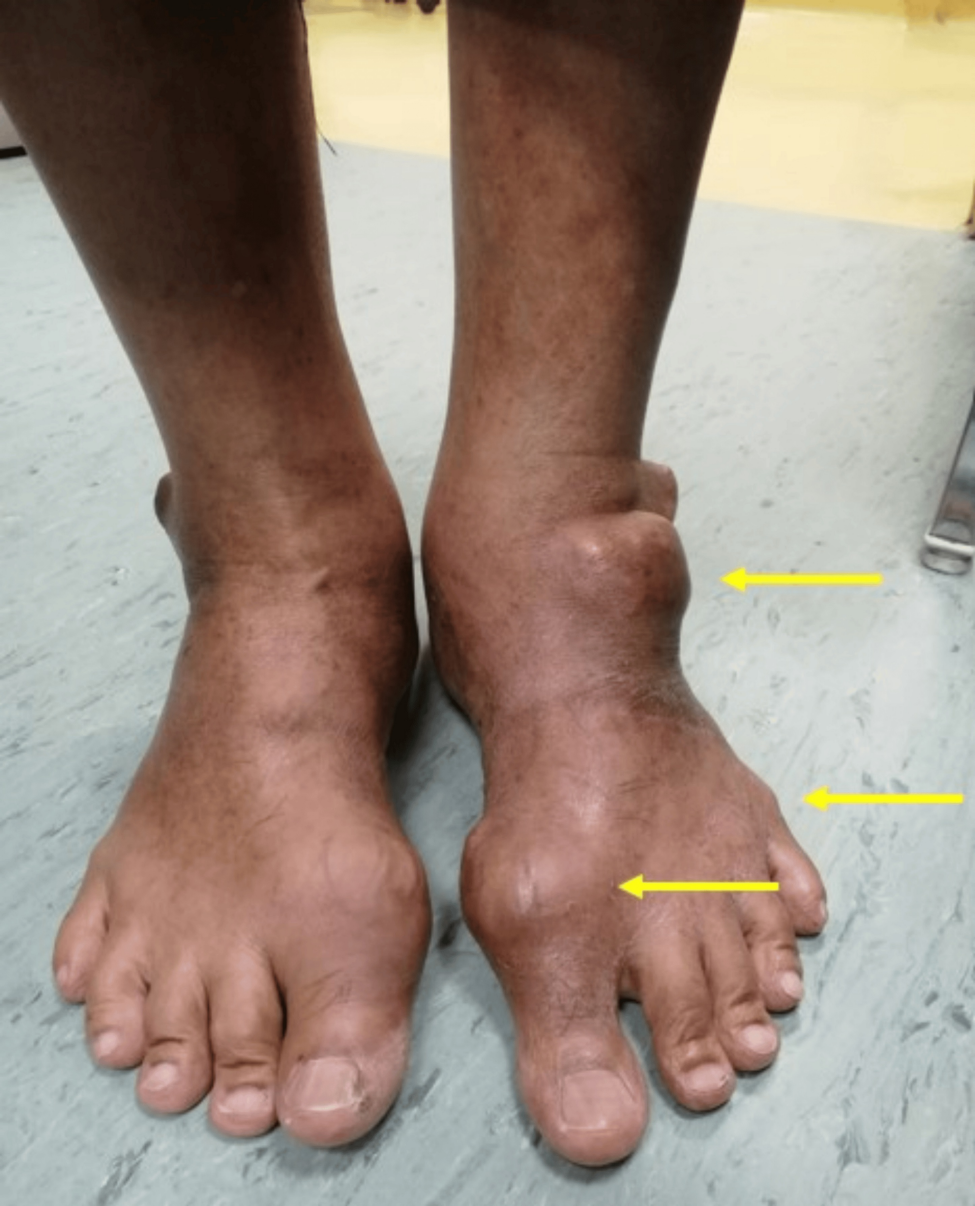
Multiple gouty tophi over ankles and chronic arthropathy of MTP joints MTP: metatarsophalangeal

Local examination revealed a wide-based, nodular mass in the right auricle's scaphoid fossa, measuring approximately 2 x 2 cm (Figure 1). The mass was rubbery in consistency, non-tender, and not warm, with a regular surface and thinned overlying skin containing whitish material. There was no palpable regional lymphadenopathy. The bilateral otoscopic examination was unremarkable. Systemic examination revealed multiple soft nodular masses of varying sizes over the bilateral fingers and ankles and firm masses over the bilateral MTP joints (Figure 2).

Biochemical investigation showed an elevated serum uric acid level of 570 µmol/L (normal range: 210-420 µmol/L). The characteristics of the auricular mass were consistent with gouty tophi, prompting the decision to proceed with an excisional mass biopsy.

**Figure 3:**
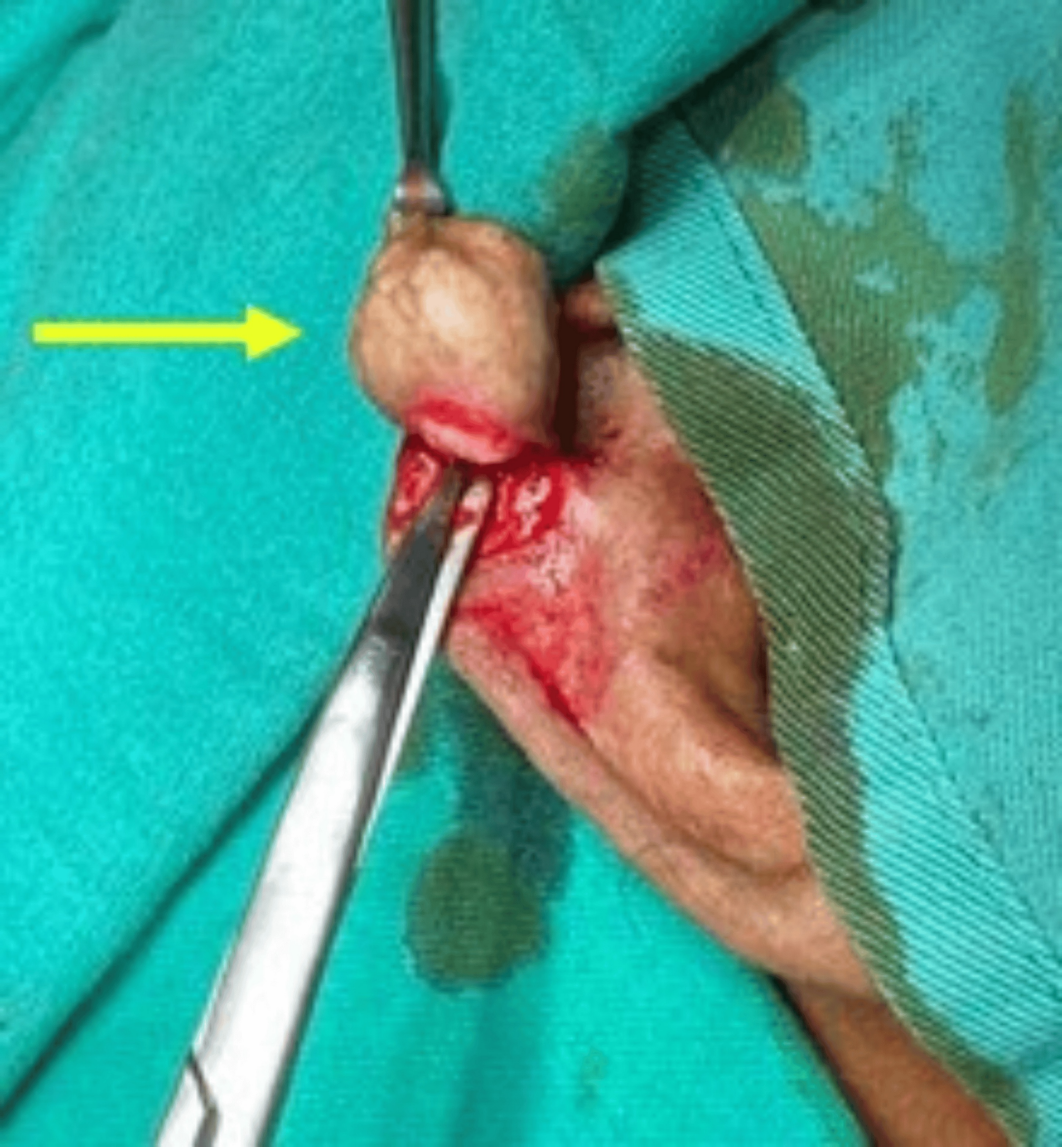
Auricular mass was completely excised with skin closed primarily

**Figure 4:**
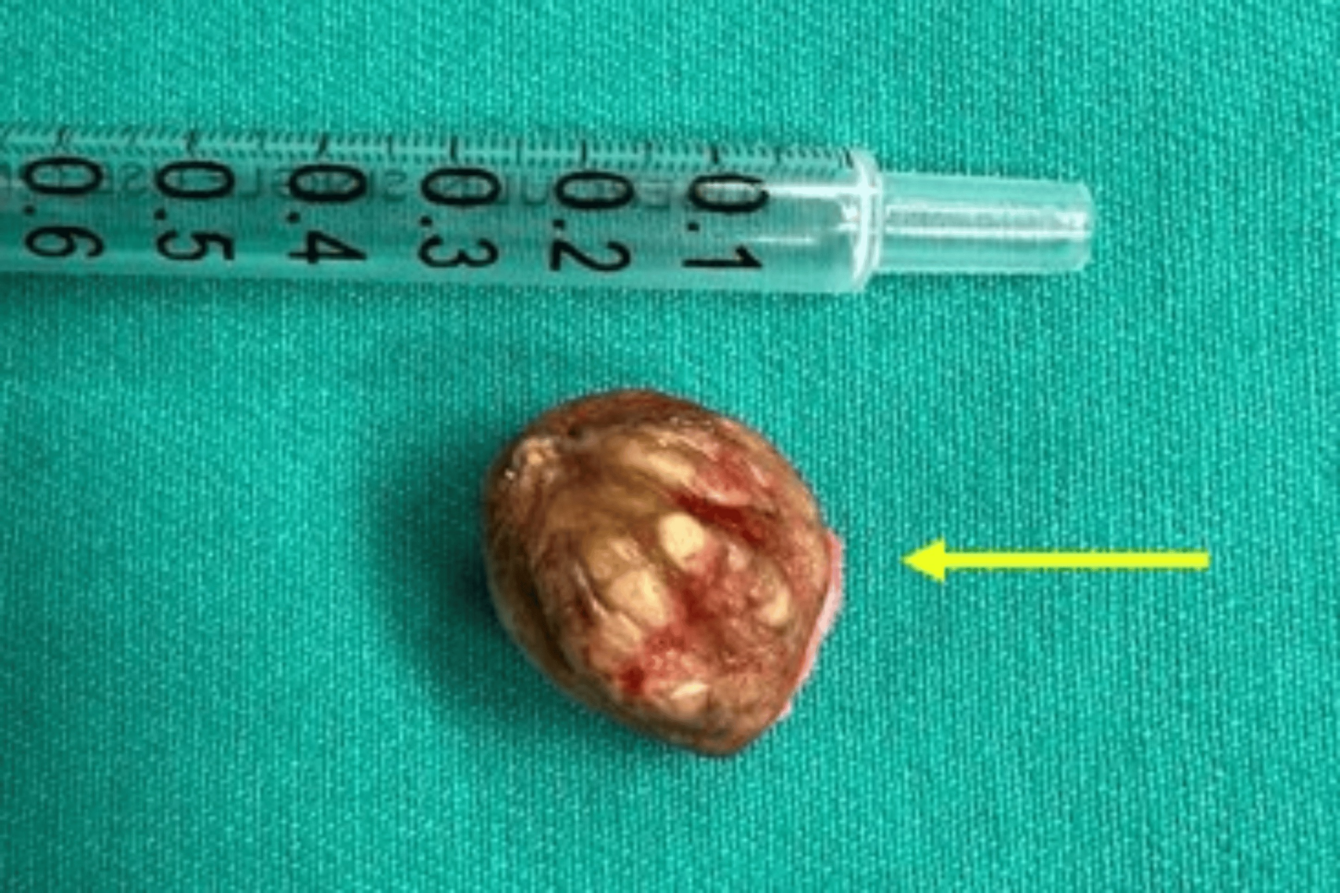
Excised auricular mass measuring approximately 2 x 2 cm

Intraoperative findings revealed a wide-based nodular mass in the right auricle's scaphoid fossa, extending its base to the helix (Figures 3-4). The mass was not attached to the underlying cartilage and had a soft to rubbery consistency, containing whitish material. The skin was undermined to allow for primary closure.

**Figure 5:**
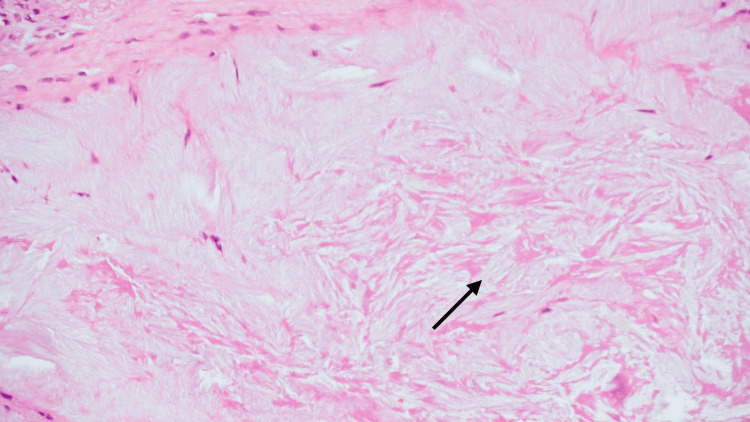
Deposition of amorphous material and needle-shaped lacunae (H&E stain, x20) H&E: hematoxylin and eosin

**Figure 6:**
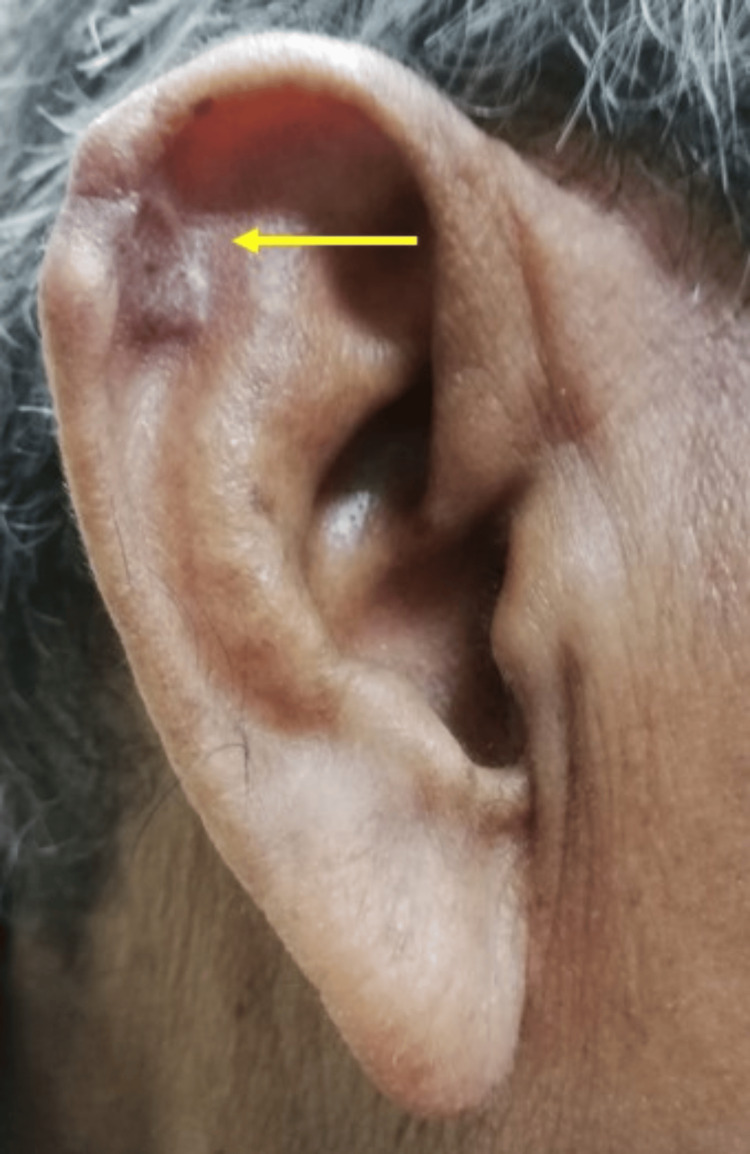
Postoperative well-healed auricular wound

The specimen was sent for histopathological examination. Microscopically, it showed fibrocollagenous tissue covered by skin, displaying multiple islands and aggregates of amorphous material consistent with monosodium urate crystal deposits (Figure 5), surrounded by lymphocytes and plasma cells as well as scattered multinucleated giant cells. At the one-month postoperative review, the auricular wound had healed well (Figure 6). Subsequent follow-up revealed a satisfactory cosmetic outcome without signs of recurrence.

## Discussion

The auricle can be affected by various skin lesions originating from the outer ear's skin, cartilage, vessels, glands, and hair follicles. The lobule (~44%) and tragus (~21%) are among the most common sites where auricular mass arises, followed by the crus of the helix (~11%) [12]. Benign lesions are locally limited to the ear, whereas malignant lesions may exhibit local invasion and potential distant metastasis [13]. Common benign auricular lesions include epidermoid cysts, hypertrophied scars, nevi, keloids, and hemangiomas [13].

The slow-growing regular auricular mass without overlying skin infiltration or regional lymph node involvement indicated a benign lesion. A nodular mass with whitish matter often raises the differential diagnosis of a sebaceous cyst. However, with the presence of concomitant uncontrolled hyperuricemia and the presence of multiple gouty tophi, auricular gouty tophi should emerge as the main differential diagnosis.

Due to the straightforward nature of the diagnosis, no additional investigations were necessary prior to excision, despite the mass's unusual location. Fine needle aspiration cytology can be useful in cases where the diagnosis is uncertain [14]. However, many auricular masses are difficult to diagnose by physical examination and fine needle aspiration [15], with most requiring surgical biopsy for definitive diagnosis.

Gouty tophi usually resolves with urate-lowering therapy, although it may take more than 30 months to significantly reduce size [16]. While larger nodules, as in this case, often necessitate surgical excision. While surgical excision of auricular gouty tophi may provide temporary relief, recurrence is possible if the serum hyperuricemia remains uncontrolled. Therefore, lowering the serum uric acid level is the mainstay of treatment through diet modification and uric acid-lowering agents, alongside surgical excision of the tophi.

## Conclusions

The diagnosis of auricular gouty tophi should be the primary consideration in patients with a nodular auricular mass, whitish material, uncontrolled hyperuricemia, and multiple gouty tophi. Although clinical history, examination findings, and biochemical investigations provide important diagnostic clues, definitive confirmation necessitates surgical excision and histopathological analysis.
